# Association of metabolic score for insulin resistance with gestational diabetes mellitus: a systematic review and meta-analysis

**DOI:** 10.3389/fnut.2026.1855104

**Published:** 2026-05-28

**Authors:** Bo Dai, Peng Zhang, Haijiao Wang, Jian Sun, Ye Zhang, Yang Xiao, Shilin Liu, Haoru Cong, Yutong Li, Xiangman He, Tan Wang, Zheng Nan, Le Liu

**Affiliations:** 1 Changchun University of Chinese Medicine, Changchun, China; 2 The Affiliated Hospital of Changchun University of Chinese Medicine, Changchun, China

**Keywords:** gestational diabetes mellitus, meta-analysis, METS-IR, risk factor, systematic review

## Abstract

**Objective:**

This study aimed to systematically evaluate the association between the Metabolic Score for Insulin Resistance (METS-IR) and the risk of gestational diabetes mellitus (GDM).

**Methods:**

A systematic search was conducted in PubMed, Embase, Web of Science, and the Cochrane Library up to March 2026. Two independent reviewers completed study screening, data extraction, and quality assessment. Cohort studies were evaluated with the Newcastle-Ottawa Scale, and cross-sectional studies were assessed with the Agency for Healthcare Research and Quality checklist. Meta-analyses were performed using Stata 16.0 and Review Manager 5.4.1. Heterogeneity was examined using the Cochrane Q-test and I^2^ statistic, and a random-effects model was used to pool effect sizes. Subgroup analyses stratified by country, study design, and sample size were conducted to explore sources of heterogeneity. Sensitivity analysis was used to validate the robustness of pooled estimates, and publication bias was assessed using funnel plots and Egger’s test.

**Results:**

A total of 6 studies involving 93,995 pregnant women were included. The overall pooled odds ratio (OR) for GDM risk in the highest versus lowest METS-IR category was 2.53 (95% CI: 2.14–3.00, I^2^ = 57%, *p* < 0.001). Subgroup analyses showed: Chinese populations had a significant positive association (OR = 2.58, 95% CI: 2.19–3.03, I^2^ = 61%, *p* < 0.001), while the association in American populations was marginally non-significant (OR = 2.80, 95% CI: 0.94–8.31, I^2^ = 71%, *p* = 0.06); Prospective cohorts (OR = 2.13, 95% CI: 1.27–3.59, I^2^ = 83%, *p* = 0.004) and retrospective cohorts (OR = 2.75, 95% CI: 2.31–3.28, I^2^ = 23%, *p* < 0.001) both showed significant positive associations, with no significant between-subgroup differences (*p* = 0.66); Large-sample studies (≥5,000 participants) had a stable, significant association (OR = 2.63, 95% CI: 2.41–2.88, I^2^ = 12%, *p* < 0.001), while small-sample studies (<5,000 participants) also showed a significant association (OR = 2.78, 95% CI: 1.41–5.47, I^2^ = 78%, *p* = 0.003). Sensitivity analyses confirmed the stability of the pooled results, and no significant publication bias was detected.

**Conclusion:**

Elevated METS-IR is significantly associated with increased GDM risk. As a noninvasive and cost-effective insulin resistance marker, METS-IR may serve as a reliable early predictor for GDM and support early screening in high-risk pregnant women. Further large-scale, multi-ethnic prospective studies are needed to validate these findings.

**Systematic review registration:**

https://www.crd.york.ac.uk/PROSPERO/view/CRD420261355910, CRD420261355910.

## 1 Introduction

Gestational diabetes mellitus (GDM) is one of the most frequent metabolic complications during pregnancy, affecting 15–20% of pregnant women worldwide with a steady upward trend in prevalence over recent years (1). GDM not only increases the risk of adverse maternal outcomes such as preeclampsia, cesarean section, and type 2 diabetes mellitus in later life, but also leads to fetal and neonatal complications including macrosomia, neonatal hypoglycemia, and long-term metabolic disorders, imposing a heavy burden on public health systems (2). Insulin resistance (IR) is recognized as the core pathophysiological mechanism underlying GDM, and early identification of IR in pregnancy is critical for the early screening, risk stratification, and timely intervention of GDM (3). However, the gold-standard hyperinsulinemic-euglycemic clamp is invasive, expensive, and impractical for population-based screening, creating an urgent demand for a convenient and reliable IR marker for GDM prediction (4).

The Metabolic Score for Insulin Resistance (METS-IR) is a newly developed indicator that combines fasting blood glucose, triglycerides, high-density lipoprotein cholesterol, and body mass index. It has been extensively confirmed as a valid and effective measure for evaluating insulin resistance in non-pregnant populations affected by type 2 diabetes, metabolic syndrome, and cardiovascular diseases (5, 6). In recent years, numerous observational investigations have examined the relationship between first-trimester METS-IR levels and the risk of GDM. However, their findings have been inconsistent among diverse populations and various study designs (7, 8). Some studies reported a significant positive dose–response relationship between elevated METS-IR and increased GDM risk, while others found limited predictive value in specific subgroups, and no systematic synthesis of the current evidence has been conducted (9). Additionally, the sources of heterogeneity across studies, such as ethnicity, study design, and sample size, have not been fully explored, which limits the clinical application of METS-IR in GDM screening.

Accordingly, this study was conducted to comprehensively assess the relationship between first-trimester METS-IR and GDM risk by integrating eligible observational studies. Subgroup analyses were performed to explore heterogeneity, and sensitivity and publication bias analyses were used to verify result robustness. This study aims to provide high-quality evidence for METS-IR as an early GDM predictive marker and support personalized GDM screening in clinical practice.

## 2 Methods

This study was registered in PROSPERO under registration number CRD420261355910 and was performed in accordance with the 2020 PRISMA guidelines (10). The corresponding PRISMA checklist is attached as Supplementary Material S1.

### 2.1 Database and search strategy

Systematic literature searches were conducted in PubMed, Embase, Web of Science, and the Cochrane Library through March 2026. Key search terms included “diabetes, gestational,” “gestational diabetes mellitus,” “GDM,” “Metabolic Score for Insulin Resistance,” “METS-IR,” and “cross-sectional study,” “case–control study,” “cohort study.” The detailed search strategy is provided in Supplementary Material S2.

### 2.2 Study selection criteria

Study selection criteria were established according to the PECOS framework.

#### 2.2.1 Participants

Pregnant women in early pregnancy, with no type 1 diabetes, type 2 diabetes, or history of GDM in prior pregnancies.

#### 2.2.2 Exposure

Studies that reported METS-IR were included. The formula for calculating METS-IR was as follows:


$$\text{METS}-\mathrm{IR}=\ln[\begin{array}{l} \left(2\times \mathrm{FBG}\;(\mathrm{mg}/\mathrm{dL})+\mathrm{TG}\;(\mathrm{mg}/\mathrm{dL})\right)\\ \times \mathrm{BMI}/\mathrm{HDL}-C\;(\mathrm{mg}/\mathrm{dL}) \end{array}]$$

#### 2.2.3 Comparators

Studies comparing the highest versus lowest quantiles of METS-IR, with a clearly defined reference group, were included.

#### 2.2.4 Outcomes

The primary outcome was newly diagnosed GDM, with clear, internationally recognized diagnostic criteria explicitly reported in each included study. Eligible studies were required to provide adjusted or unadjusted odds ratio (OR) with corresponding 95% confidence interval (CI) for the association between METS-IR and the risk of newly diagnosed GDM.

#### 2.2.5 Study design

Eligible studies were observational investigations, encompassing cohort, case–control, and cross-sectional designs, that were published in English in peer-reviewed journals with accessible full texts and focused on the association between METS-IR and GDM risk.

### 2.3 Exclusion criteria

#### 2.3.1 Types of participants

Participants were excluded if they had pregestational diabetes mellitus, multiple pregnancies, chronic liver or kidney disease, endocrine disorders (including thyroid dysfunction), or were receiving medications known to affect glucose or lipid metabolism.

#### 2.3.2 Exposure

Studies using other metabolic indices or measuring METS-IR in the second or third trimester were excluded.

#### 2.3.3 Comparators

Studies without a clearly defined reference group, or using arbitrary cut-offs not validated for GDM prediction, were excluded.

#### 2.3.4 Outcome measures

Studies reporting only other pregnancy outcomes without GDM data, or using non-standard GDM diagnostic criteria, were excluded.

#### 2.3.5 Type of study type

Case reports, reviews, meta-analyses, letters, editorials, and conference abstracts were excluded, as were studies with insufficient data to calculate OR and 95% CI.

### 2.4 Literature screening and data extraction

Two researchers (ZP, WHJ) constructed the literature database using EndNote X9 software. They employed cross-validation to confirm the inclusion and exclusion criteria for studies. Disagreements were arbitrated by a third researcher (DB), with final consensus reached through discussion. Two additional researchers (LSL, CHR) independently extracted key information from the included studies, including Study, Country, Study design, Gestational trimester, Age, Number of participants, METS-IR, OR (95% CI), Outcome type, and Variables adjusted. Data extraction was validated through cross-checking.

### 2.5 Quality assessment

Two independent researchers (SJ, DB) independently assessed the methodological quality of all included studies. Cohort studies were evaluated using the Newcastle-Ottawa Scale (NOS), covering three dimensions: participant selection, comparability of study groups, and outcome assessment (11). The total NOS score ranges from 0 to 9, with studies scoring 7 or higher defined as high-quality. Cross-sectional studies were appraised using the Agency for Healthcare Research and Quality (AHRQ) checklist (12). Any disagreements between the two investigators were resolved via discussion until a consensus was achieved.

### 2.6 Statistical analysis

Statistical analyses for GDM outcomes were conducted using Review Manager 5.4.1 and Stata 16 software. The OR with 95% CI were applied as effect indicators to evaluate the relationship between METS-IR and GDM risk. For categorical METS-IR, pooled OR were estimated by comparing the highest with the lowest categories or quartiles.

Heterogeneity across studies was evaluated by the Cochrane Q-test with a significance level of *p* < 0.1. The I^2^ statistic with 95% CI was used to quantify the magnitude of heterogeneity. The interpretation of I^2^ was based on the Cochrane Handbook for Systematic Reviews of Interventions (7th edition): values of 0–40% represented low or no heterogeneity, 30–60% represented moderate heterogeneity, 50–90% represented substantial heterogeneity, and 75–100% represented considerable heterogeneity (13). Subgroup analyses stratified by country, study design, and sample size were performed to identify potential sources of heterogeneity. Pooled OR were calculated for each subgroup, and between-subgroup differences were examined using the Q-test with *p* < 0.05 defined as statistically significant. A fixed-effects model was adopted when heterogeneity was low (*p* > 0.1 and I^2^ ≤ 40%). If significant heterogeneity was present (*p* < 0.1 or I^2^ ≥ 50%), predefined subgroup analyses were first conducted to explore sources of variation, and a random-effects model was then used for the pooled analysis.

Sensitivity analysis was performed to examine the stability of the main results by omitting each study individually and recalculating the combined OR. Funnel plots and Egger’s test were adopted to evaluate potential publication bias, and *p* < 0.05 was considered statistically significant.

## 3 Results

### 3.1 Literature screening results

**Figure 1:**
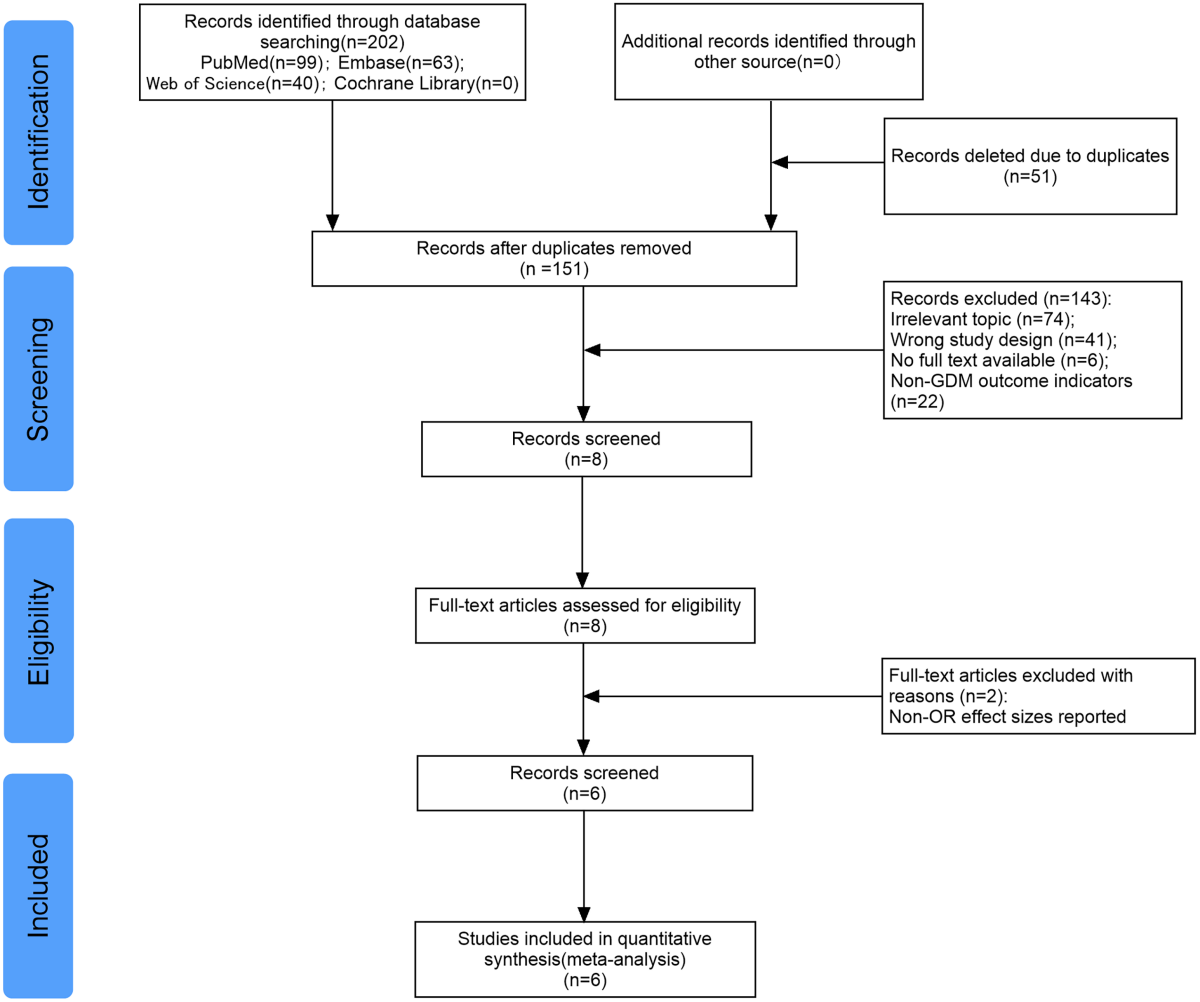
Flowchart of study selection.

A total of 202 records were initially retrieved. Following the removal of 51 duplicate entries, 143 records were excluded after title and abstract screening. The remaining studies were subjected to full-text assessment, with 2 studies excluded due to the reporting of non-OR effect sizes. Ultimately, 6 studies were included in this study (7, 8, 14–17). The literature screening process is depicted in Figure 1.

### 3.2 Basic information of the included studies

All studies included in the present meta-analysis were published between 2025 and 2026, with individual sample sizes varying from 472 to 46,992 participants. The combined sample size across all eligible studies amounted to 93,995, and the detailed characteristics of each study are summarized in Table 1.

**Table 1:** Basic characteristics of the included studies.

Number	Study	Country	Age (year)	Number of participants	Gestational trimester	METS-IR condition	OR (95%CI)	Study design	Type of outcome	Variables adjusted
1	Wenxuan et al (2025) (7)	America	Average age: 52.86 ± 16.48 years	5,189	—	Categorized	1.76 (1.03–3.00)	Cross-Sectional study	Gestational Diabetes Mellitus	Age, race, education level, the ratio of family PIR, high blood pressure, total cholesterol, smoking, physical activity, sedentary duration and A history of delivering an infant weighing 9 pounds or more.
2	Gao et al. (2025) (14)	China	Median age: 31 years	1,450	First trimester (<12 weeks)	Categorized	1.573 (1.047–2.373)	Prospective cohort study	Gestational Diabetes Mellitus	Age, prepregnant weight gain rate, diastolic blood pressure, fasting insulin, Low-density lipoprotein cholesterol and Family history of diabetes mellitus.
3	Zhang et al. (2025) (15)	China	Average age: GDM: 34.52 ± 3.97 years No GDM: 33.09 ± 3.83 years	46,992	First trimester (6–13 + 6 weeks)	Categorized	2.69 (2.33–3.10)	Prospective cohort study	Gestational Diabetes Mellitus	Age, multiparity, pre-pregnancy BMI, family history of diabetes, history of GDM, education level, and first-trimester CHO and low density lipoprotein.
4	Pan et al. (2026) (16)	China	Average age: GDM: 30.90 ± 4.72 years No GDM: 29.33 ± 4.43 years	2,122	First trimester (4–8 weeks)	Categorized	3.41 (2.23–5.21)	Retrospective cohort study	Gestational Diabetes Mellitus	Age, systolic blood pressure, diastolic blood pressure, total bilirubin, direct bilirubin, alanine transaminase, blood urea nitrogen, low density ipoprotein, total cholesterol and uric acid.
5	Li et al. (2025) (17)	China	Average age: 30.76 ± 3.87 years	37,770	—	Categorized	2.65 (2.42–2.91)	Retrospective cohort study	Gestational Diabetes Mellitus	Age, test week, creatinine, alanine aminotransferase, total cholesterol, hypertension history, diabetes history, tobacco, alcohol, IVF, adverse pregnancy history, parity and education levels.
6	Dong and Zhang (2026) (8)	America	Average age: 28.88 ± 5.84 years	472	—	Categorized	5.43 (1.87–15.75)	Cross-Sectional study	Gestational Diabetes Mellitus	Age, race, educational level, marital status, income-to-poverty ratio, body mass index, smoking status, alcohol drinking, hypertension, low-density lipoprotein cholesterol, triglyceride, high-density lipoprotein cholesterol, alanine aminotransferase, aspartate aminotransferase and serum creatinine.

### 3.3 Quality assessment

Methodological quality evaluation of the included studies was conducted using the NOS for cohort studies and the AHRQ checklist for cross-sectional studies. The NOS evaluates three dimensions: cohort selection, group comparability, and outcome measurement, employing a binary scoring system (1 point for meeting criteria, 0 for not meeting them) with a maximum total score of 9. The AHRQ instrument consists of 11 items, with a highest attainable score of 11. All four cohort studies achieved a full score of 9, whereas the two cross-sectional studies scored 10 and 9 points, respectively. These results confirmed the overall high methodological quality of all six included studies, and the detailed quality assessment data are displayed in Tables 2, 3.

**Table 2:** Details of quality evaluation via the Newcastle–Ottawa scale.

Study (publication year)	Selection of cohorts			Comparability of cohorts			Outcome of cohorts			Total
	Representativeness of the exposed cohort	Selection of the non-exposed cohort	Ascertainment of exposure	Outcome not present at baseline	Control for age	Control for other confounding factors	Assessment of outcome	Sufficient follow-up duration	Adequacy of follow-up of cohorts	
Gao et al. (2025) (14)	1	1	1	1	1	1	1	1	1	9
Zhang et al. (2025) (15)	1	1	1	1	1	1	1	1	1	9
Pan et al. (2026) (16)	1	1	1	1	1	1	1	1	1	9
Li et al. (2025) (17)	1	1	1	1	1	1	1	1	1	9

**Table 3:** Details of quality evaluation via the agency for healthcare research and quality checklist.

Study (publication year)	D1	D2	D3	D4	D5	D6	D7	D8	D9	D10	D11	Overall
Wenxuan et al (2025) (7)	1	1	1	1	1	1	1	1	1	9	0	10
Dong and Zhang (2026) (8)	1	1	1	1	1	1	1	1	0	1	0	9

D1: Clear definition of study population; D2: Clear inclusion/exclusion criteria; D3: Clear description of data collection; D4: Standardized exposure measurement; D5: Standardized outcome measurement; D6: Adjustment for key confounders; D7: Representative sampling frame; D8: Low risk of selection bias; D9: Low risk of measurement/information bias; D10: Appropriate statistical methods; D11: Reported study limitations.

### 3.4 Meta-analysis results

**Figure 2:**
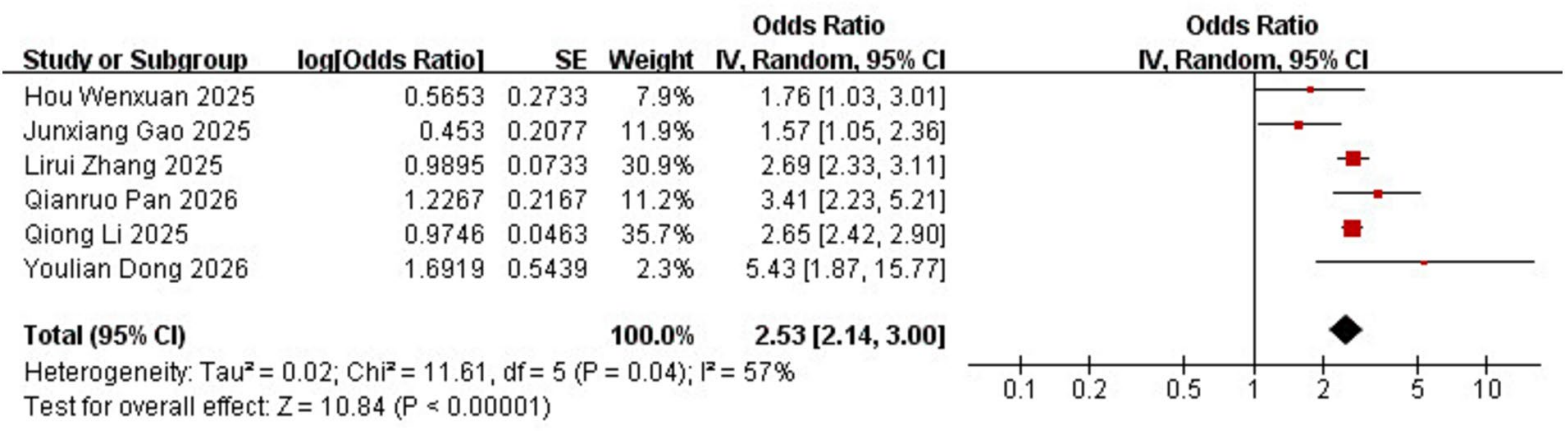
Forest plot of the association between METS-IR and GDM risk.

A total of 6 observational studies encompassing 93,995 participants were incorporated into this meta-analysis. The overall pooled OR was 2.53 (95% CI: 2.14–3.00, I^2^ = 57%; *p* < 0.001), which suggested a significant positive association between elevated METS-IR levels and a higher risk of GDM. All included studies exhibited a consistent direction of effect, alongside moderate between-study heterogeneity (I^2^ = 57%, *p* = 0.04). A random-effects model was adopted for effect size pooling. The forest plot is presented in Figure 2.

**Figure 3:**
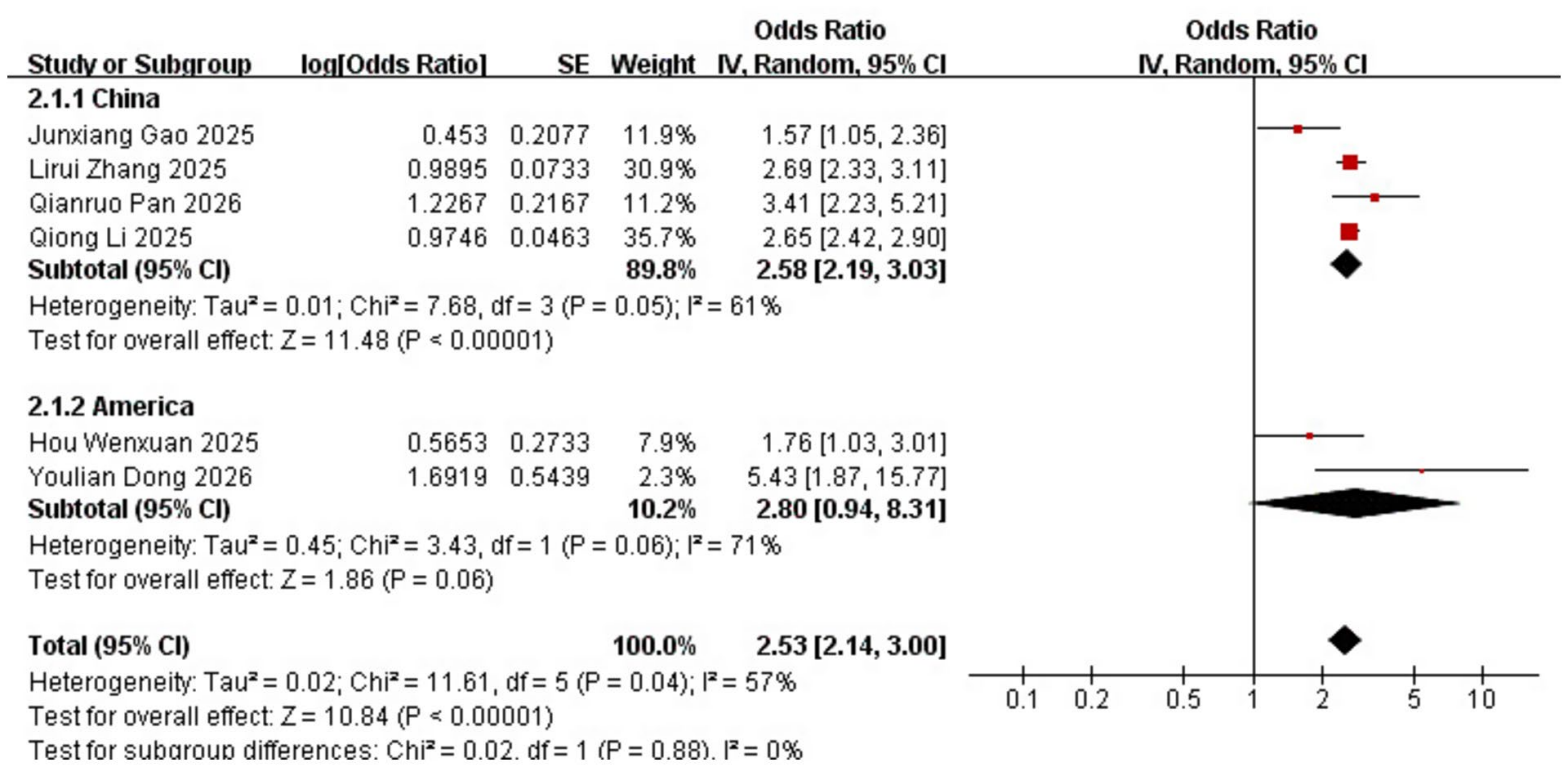
Subgroup analysis by country.

**Figure 4:**
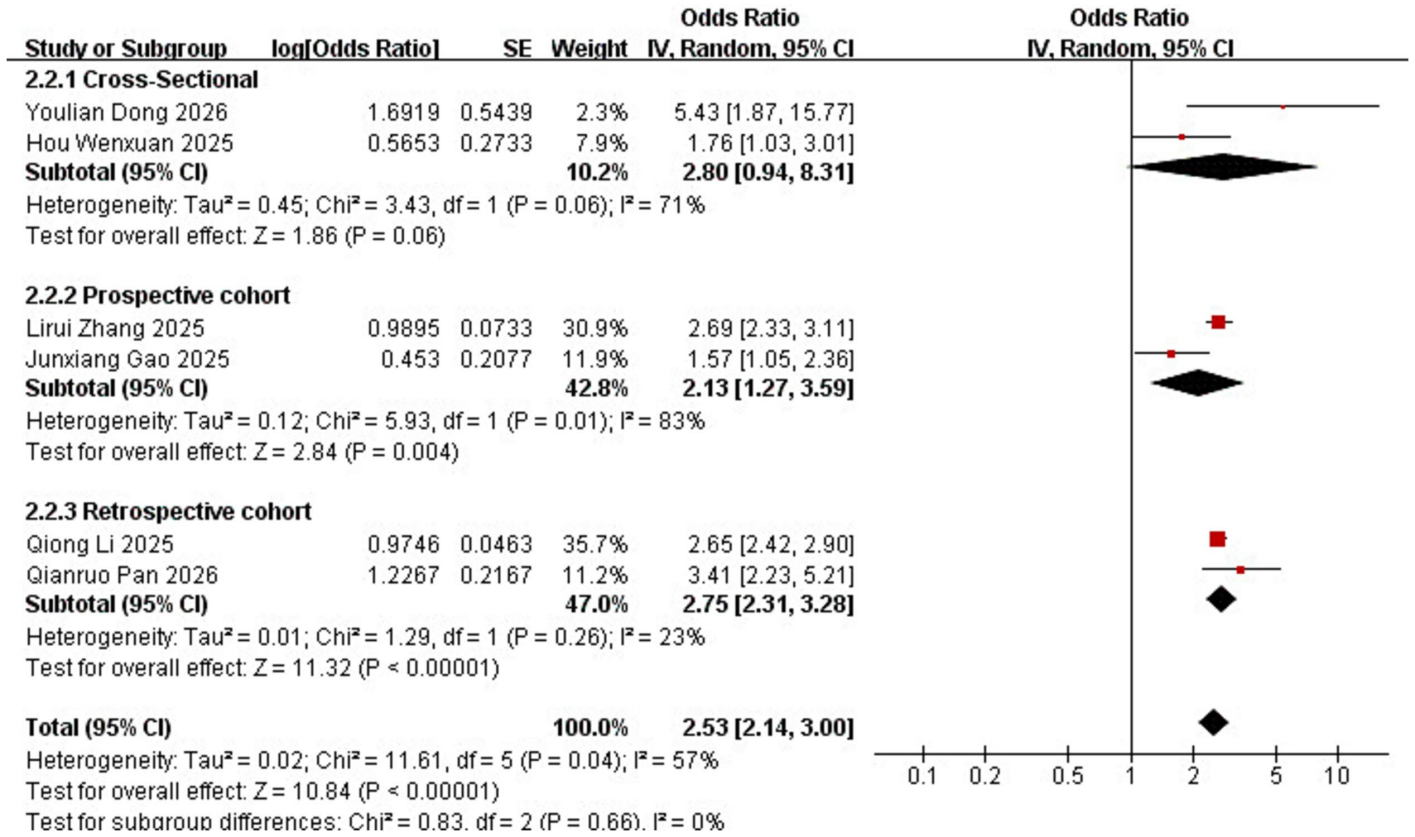
Subgroup analysis by study design.

**Figure 5:**
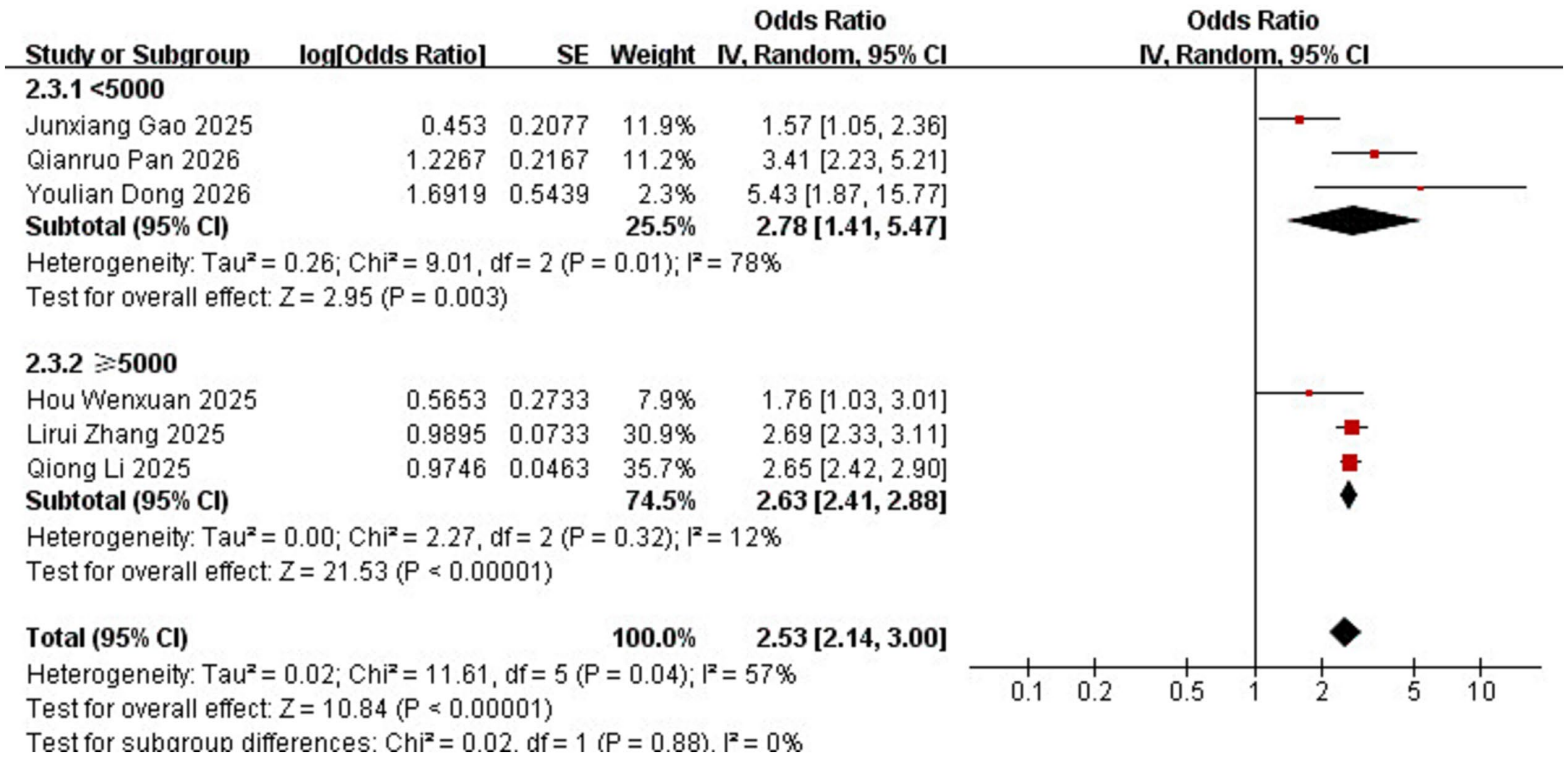
Subgroup analysis by sample size.

To investigate potential sources of heterogeneity, subgroup analyses were performed based on country, study design, and sample size. When stratified by country, the Chinese population subgroup had a pooled OR of 2.58 (95% CI: 2.19–3.03, *p* < 0.001) with moderate heterogeneity (I^2^ = 61%). The American population subgroup showed a pooled OR of 2.80 (95% CI: 0.94–8.31, *p* = 0.06) with high heterogeneity (I^2^ = 71%). No significant between-subgroup difference was detected (χ^2^ = 0.02, df = 1, *p* = 0.88; I^2^ = 0%). The corresponding forest plots are shown in Figure 3. For subgroup analysis by study design, the cross-sectional subgroup yielded a pooled OR of 2.80 (95% CI: 0.94–8.31, *p* = 0.06) with high heterogeneity (I^2^ = 71%). The prospective cohort subgroup had a pooled OR of 2.13 (95% CI: 1.27–3.59, *p* = 0.004) with high heterogeneity (I^2^ = 83%), whereas the retrospective cohort subgroup presented a pooled OR of 2.75 (95% CI: 2.31–3.28, *p* < 0.001) with low heterogeneity (I^2^ = 23%). No significant difference was observed across subgroups (χ^2^ = 0.83, df = 2, *p* = 0.66; I^2^ = 0%). The relevant forest plots are displayed in Figure 4. When stratified by sample size, the large-sample subgroup (≥5,000 participants) had a pooled OR of 2.63 (95% CI: 2.41–2.88, p < 0.001) with low heterogeneity (I^2^ = 12%). The small-sample subgroup (<5,000 participants) showed a pooled OR of 2.78 (95% CI: 1.41–5.47, *p* = 0.003) with high heterogeneity (I^2^ = 78%). No significant between-subgroup difference was found (χ^2^ = 0.02, df = 1, *p* = 0.88; I^2^ = 0%). The corresponding forest plots are illustrated in Figure 5.

### 3.5 Publication bias analysis

**Figure 6:**
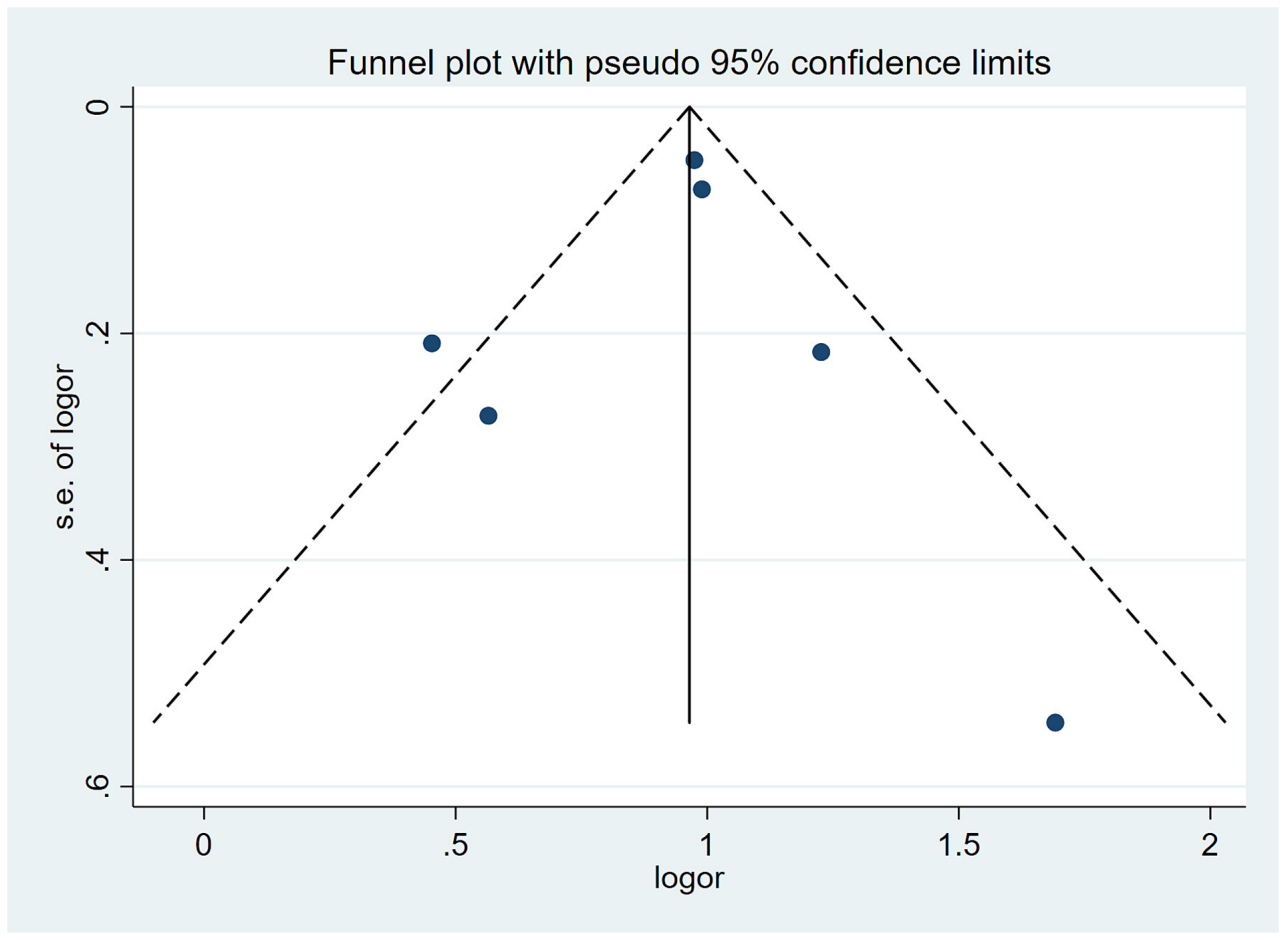
Funnel plot for publication bias.

Publication bias was assessed using both funnel plot inspection and formal statistical tests. Begg’s rank correlation test showed no significant publication bias (z = 0.94, *p* = 0.348; continuity-corrected z = 0.75, *p* = 0.452). Egger’s linear regression test further confirmed the absence of significant bias, with a non-significant intercept coefficient of −0.3357 (SE = 1.0735, t = −0.31, *p* = 0.770, 95% CI: −3.3162 to 2.6447). All statistical tests yielded *p*-values > 0.05, indicating no evidence of publication bias. Visual inspection of the funnel plot was consistent with these findings, showing symmetric distribution of the included studies around the pooled effect estimate. It should be noted, however, that the reliability of publication bias assessment is limited when fewer than 10 studies are included. Therefore, the results of these tests should be interpreted with extreme caution, and the possibility of undetected publication bias cannot be entirely ruled out. The corresponding funnel plot is presented in Figure 6.

### 3.6 Sensitivity analysis

A leave-one-out sensitivity analysis was performed to assess the stability of the pooled effect estimate. Exclusion of any single study from the meta-analysis yielded pooled OR ranging from 2.44 to 2.68, all of which remained statistically significant (*p* < 0.001). The heterogeneity fluctuated slightly across the analyses, ranging from 25 to 65%, without substantial changes in the overall effect direction or magnitude. Exclusion of each individual study did not markedly alter the overall pooled effect size or the statistical significance of the association. These findings verify the robustness of the overall association between higher METS-IR levels and elevated GDM risk, given that the direction, magnitude, and statistical significance of the results remained consistent across all sensitivity analyses. Detailed outcomes of the sensitivity analysis are presented in Table 4.

**Table 4:** Summary of sensitivity analysis.

Dataset excluded	OR	95%CI	I^2^%	P for effect
Wenxuan et al (2025) (7)	2.62	[2.21, 3.09]	58	*p* < 0.001
Gao et al. (2025) (14)	2.68	[2.40, 3.00]	25	*p* < 0.001
Zhang et al. (2025) (15)	2.44	[1.81, 3.30]	65	*p* < 0.001
Pan et al. (2026) (16)	2.44	[2.04, 2.92]	60	*p* < 0.001
Li et al. (2025) (17)	2.45	[1.79, 3.36]	65	*p* < 0.001
Dong and Zhang (2026) (8)	2.50	[2.12, 2.94]	59	*p* < 0.001

## 4 Discussion

This meta-analysis comprehensively evaluated the association between first-trimester METS-IR and the risk of GDM by including 6 high-quality observational studies involving 93,995 participants. The main finding demonstrated that elevated METS-IR levels were significantly associated with an increased risk of GDM, with a pooled OR of 2.53 (95% CI: 2.14–3.00, *p* < 0.001). To our knowledge, this is the first systematic review and meta-analysis focusing on the predictive value of METS-IR for GDM, providing robust evidence for early risk stratification in clinical practice.

Moderate between-study heterogeneity (I^2^ = 57%) was detected in the overall analysis, which may stem from differences in ethnicity, study design, sample size, GDM diagnostic criteria, METS-IR cutoff values, and confounding adjustment strategies across studies. Such variations can influence metabolic phenotypes and insulin resistance dynamics, contributing to inter-study variability. Despite the presence of moderate heterogeneity, the consistent direction and significance of the association across all subgroups support the reliability and stability of the main findings.

The methodological quality of all included studies was high. Four cohort studies achieved full scores on the NOS, and two cross-sectional studies scored 9 and 10 points using the AHRQ checklist, respectively. Sensitivity analysis confirmed that omission of any single study did not reverse the direction or significance of the pooled effect, with ORs ranging from 2.44 to 2.68 (all *p* < 0.001). No significant publication bias was detected by funnel plot visualization, Begg’s test, and Egger’s test. These results collectively support the stability and reliability of the conclusions.

Potential biological mechanisms may explain the positive association between METS-IR and GDM risk. First, METS-IR reflects systemic insulin resistance by integrating glucose, lipid metabolism, and adiposity indicators, all of which are core drivers of pancreatic *β*-cell compensation failure in early pregnancy (18). Insulin resistance impairs insulin-mediated glucose uptake in peripheral tissues such as skeletal muscle and adipose tissue, leading to compensatory hyperinsulinemia and gradual β-cell exhaustion, which further promotes the development of GDM (19). Second, elevated triglycerides and reduced high-density lipoprotein cholesterol included in METS-IR indicate dyslipidemia, which induces oxidative stress, chronic low-grade inflammation, and endoplasmic reticulum stress in pancreatic islets and placenta, further aggravating insulin resistance and impairing glucose homeostasis (20). Third, obesity reflected by higher BMI contributes to adipose tissue dysfunction and excessive release of free fatty acids and proinflammatory cytokines, all of which disrupt insulin signaling and increase GDM susceptibility (21).

Notably, the mTOR signaling pathway serves as a central mechanistic link connecting abnormal METS-IR profiles and the onset and progression of GDM (22). As an evolutionarily conserved nutrient-sensing and metabolic regulatory hub, the mTOR pathway dynamically integrates metabolic signals derived from circulating glucose, lipid profiles, and overall body adiposity—all core constituent components of the METS-IR index. Excessive and sustained mTOR hyperactivation can markedly suppress downstream insulin signaling transduction, diminish peripheral insulin sensitivity, impair pancreatic *β*-cell proliferation, differentiation and insulin secretory capacity, disrupt placental lipid homeostasis and nutrient transport function, as well as exacerbate chronic low-grade inflammatory responses in adipose and placental tissues. Collectively, these molecular and pathological alterations synergistically accelerate the underlying pathogenesis and progression of GDM (23, 24). Such underlying mechanistic evidence further supports that elevated METS-IR can function as a robust and comprehensive surrogate marker reflecting subclinical metabolic dysregulation that occurs months prior to the clinical manifestation of GDM.

**Figure 7:**
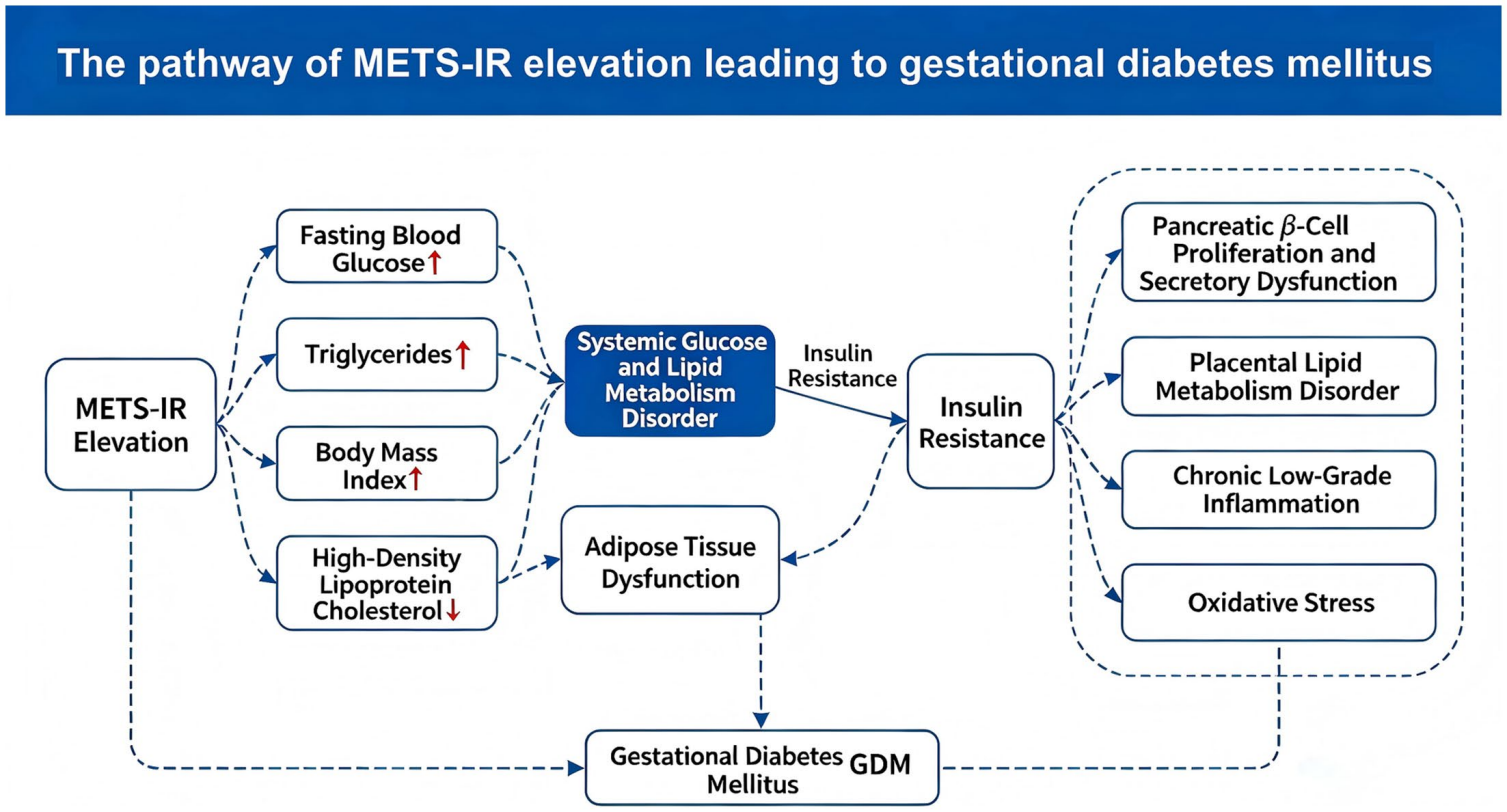
Schematic diagram of the potential mechanism linking elevated first-trimester METS-IR to the development of GDM.

To more intuitively illustrate the underlying pathological pathway, we constructed a schematic model summarizing the sequential relationship between METS-IR and GDM. Elevated first-trimester METS-IR contributes to systemic glycolipid metabolism disorder and adipose tissue dysfunction, further inducing peripheral insulin resistance. On this basis, impaired pancreatic *β*-cell secretory function, disturbed placental lipid homeostasis, sustained chronic inflammation, and oxidative stress jointly accelerate the onset of GDM. This conceptual pathway visually clarifies how METS-IR acts as an early predictive biomarker mediating metabolic dysfunction and subsequent GDM risk. The potential mechanistic pathway from elevated METS-IR to insulin resistance and subsequent GDM development is illustrated in Figure 7.

GDM is a common metabolic disorder during pregnancy characterized by insulin resistance and pancreatic β-cell dysfunction, which increases the risk of adverse maternal and neonatal outcomes (25). Traditional insulin resistance indices such as HOMA-IR require insulin detection, limiting their application in early pregnancy screening (26). As a non-insulin-based indicator, METS-IR integrates fasting blood glucose, triglycerides, body mass index, and high-density lipoprotein cholesterol, which are routinely measured in early pregnancy (27). Our results confirmed that higher METS-IR was an independent risk factor for GDM, supporting the notion that METS-IR can serve as a convenient and reliable biomarker for early identification of women at high GDM risk.

## 5 Strengths and limitations

This meta-analysis has several notable strengths. First, this study is the first meta-analysis to explore the association between early-pregnancy METS-IR and the risk of GDM, providing high-quality evidence for clinical risk stratification. Second, all included studies were of high methodological quality; cohort studies achieved full NOS scores, and cross-sectional studies scored highly on the AHRQ checklist. Third, the pooled sample size was large, and the leave-one-out sensitivity analysis confirmed the robustness and stability of the pooled effect. Fourth, no significant publication bias was detected by funnel plot, Begg’s test, or Egger’s test, supporting the reliability of the conclusions. Finally, subgroup analyses by country, study design, and sample size yielded consistent results, further reinforcing the credibility of the findings.

Several limitations of this meta-analysis should also be acknowledged. First, moderate between-study heterogeneity (I^2^ = 57%) was observed, which may stem from differences in baseline characteristics, GDM diagnostic criteria, METS-IR cutoff values, and adjusted confounding factors across studies. Second, all included studies were conducted in Chinese and American populations, which may restrict the generalizability of the results to other ethnic or geographic regions. Third, individual participant data were not available, limiting further adjustment for key confounders such as diet, physical activity, and family history of diabetes. In addition, only six studies were included in this meta-analysis; although all were high-quality investigations with a large combined sample size (93,995 participants) and consistent effect directions, the small number of included studies may still reduce statistical power and limit further subgroup stratification, potentially decreasing the precision of pooled effect estimates. Therefore, caution is warranted when generalizing the conclusions, and future large-scale, multi-ethnic prospective cohort studies with standardized protocols are urgently needed to validate the robustness and generalizability of the present findings.

## 6 Conclusion

In conclusion, elevated METS-IR levels in early pregnancy are significantly associated with an increased risk of GDM. As a simple, non-insulin-dependent, and easily obtainable metabolic index, METS-IR demonstrates valuable potential for the early screening and risk stratification of GDM. Routine assessment of METS-IR in the first trimester could help identify high-risk women and facilitate timely interventions, thereby reducing the incidence of GDM and its adverse pregnancy outcomes.

## Data Availability

The datasets presented in this study can be found in online repositories. The names of the repository/repositories and accession number(s) can be found in the article/Supplementary material.
